# Potential Involvement of Myostatin in Smooth Muscle Differentiation in Pleomorphic Leiomyosarcoma

**DOI:** 10.3390/ijms26167676

**Published:** 2025-08-08

**Authors:** Hiroko Onagi, Raku Son, Akiko Oguchi, Kei Sano, Keita Sasa, Nobuhiko Hasegawa, Keisuke Akaike, Daisuke Kubota, Tatsuya Takagi, Takuo Hayashi, Muneaki Ishijima, Takashi Yao, Yoshiyuki Suehara, Yasuhiro Murakawa, Tsuyoshi Saito

**Affiliations:** 1 Department of Human Pathology, Juntendo University School of Medicine, Tokyo 113-8421, Japan; h-onagi@juntendo.ac.jp (H.O.); k-sasa@juntendo.ac.jp (K.S.); tkhyz@juntendo.ac.jp (T.H.); tyao@juntendo.ac.jp (T.Y.); 2 RIKEN Center for Integrative Medical Sciences, Yokohama 230-0045, Japan; raku.son@riken.jp (R.S.); akiko.oguchi@riken.jp (A.O.); yasuhiro.murakawa@riken.jp (Y.M.); 3 Department of Medicine for Orthopaedics and Motor Organ, Juntendo University School of Medicine, Tokyo 113-8421, Japan; ksano@juntendo.ac.jp (K.S.); n-hasegawa@juntendo.ac.jp (N.H.); k-akaike@juntendo.ac.jp (K.A.); dkubota@juntendo.ac.jp (D.K.); ttatsuya@juntendo.ac.jp (T.T.); ishijima@juntendo.ac.jp (M.I.); ysuehara@juntendo.ac.jp (Y.S.); 4 Institute for the Advanced Study of Human Biology (ASHBi), Kyoto University, Kyoto 606-8501, Japan; 5 IFOM-The FIRC Institute of Molecular Oncology, 20139 Milan, Italy; 6 Intractable Disease Research Center, Juntendo University Graduate School of Medicine, Tokyo 113-8421, Japan

**Keywords:** pleomorphic leiomyosarcoma, myostatin, cap analysis of gene expression, double immunostaining, smooth muscle differentiation

## Abstract

High-grade sarcomas often lack typical morphological features and exhibit no clear differentiation, often leading to a diagnosis of undifferentiated sarcoma (US). Pleomorphic leiomyosarcoma (PLMS) is a high-grade sarcoma consisting of a typical leiomyosarcoma (LMS) component alongside dedifferentiated high-grade areas. A few decades ago, PLMS was regarded as a subtype of high-grade sarcoma previously referred to as malignant fibrous histiocytoma; it is now classified as a variant of LMS. The mechanisms underlying myogenic differentiation and their relevance to the pathological diagnosis of high-grade sarcomas remain poorly understood. To investigate the gene expression networks associated with myogenic differentiation, we employed Cap Analysis of Gene Expression (CAGE) to distinguish PLMS from other high-grade sarcoma subtypes. We analyzed 27 frozen high-grade sarcoma samples, comprising 10 PLMSs, 11 high-grade myxofibrosarcomas, 3 dedifferentiated liposarcomas, 2 USs, and 1 high-grade sarcoma not otherwise specified, using CAGE profiling. Hierarchical clustering based on differentially expressed genes identified by CAGE separated 7 of the 10 PLMSs from other high-grade sarcomas, while the remaining 3 PLMSs clustered with a single US case. CAGE analysis also revealed that the myostatin (*MSTN*) promoter (false discovery rate [FDR] < 0.05) was more strongly activated in the high-grade sarcoma group lacking morphological and immunohistochemical smooth muscle differentiation than in the PLMS group, whereas the alpha smooth muscle actin (*ACTA2*) promoter (FDR < 0.05) was more prominently activated in the PLMS group. Immunohistochemical analysis showed reduced or absent myostatin expression in PLMSs, in contrast to diffuse myostatin expression in other high-grade sarcomas. Smooth muscle actin, encoded by *ACTA2*, was expressed in all 10 PLMS cases but only in 11 of 17 other high-grade sarcomas. Furthermore, both conventional immunohistochemistry and double immunostaining revealed that myostatin and myogenic markers exhibited largely mutually exclusive expression patterns within these tumors. A validation study was performed using 59 soft tissue sarcoma cases, including 27 PLMSs and 16 LMSs. Loss or reduction in myostatin expression was confirmed in both LMS and PLMS, and the ratio of myostatin loss was comparable (62.5% in LMS vs. 63% in PLMS). Collectively, these findings suggest that myostatin contributes to smooth muscle differentiation in high-grade sarcomas and has potential utility as a diagnostic marker.

## 1. Introduction

Soft tissue sarcomas (STSs) are a heterogeneous group of malignant tumors [1]. Based on their malignant potential, sarcomas are classified as low-grade, intermediate-grade, or high-grade. Among high-grade sarcomas, undifferentiated pleomorphic sarcoma (UPS), previously known as malignant fibrous histiocytoma (MFH), was considered one of the most common STS subtypes until the early 2000s [1]. Over the years, however, the concept of fibrohistiocytic differentiation has been challenged [2]. Studies have shown that the MFH phenotype more closely resembles fibroblasts than histiocytes [2,3]. In 2013, MFH was removed from the World Health Organization (WHO) classification of STSs. Today, UPS is diagnosed only after excluding any identifiable feature of differentiation using currently available diagnostic technologies [4,5].

Leiomyosarcoma (LMS) is a malignant neoplasm characterized by fascicular proliferation of spindle cells with hyperchromatic nuclei and abundant eosinophilic cytoplasm, along with immunohistochemically detected smooth muscle differentiation [4]. Pleomorphic leiomyosarcoma (PLMS) is defined as a high-grade sarcoma consisting of typical LMS tissue, usually accompanied by a dedifferentiated area. This dedifferentiated component expands substantially, sometimes reducing the conventional LMS features to less than 5% [4,6]. PLMS accounts for approximately 8% of all soft tissue LMSs [7,8,9]. Immunohistochemistry (IHC) has identified significant subsets of PLMSs—previously known as MFHs—distinguishing them from UPSs [10]. Clinically, PLMS is associated with a poorer prognosis than conventional LMS and a similar prognosis to that of UPS [10]. In the absence of evident muscular differentiation as defined by immunohistochemical expression of smooth muscle markers, LMS, particularly PLMS, may otherwise be misclassified as UPS. Comprehensive genomic analysis has revealed that soft tissue LMSs typically exhibit low levels of copy number alterations (CNAs) and mutations, while UPSs show high CNA levels and low mutation burdens [11]. Furthermore, the methylation profile can reliably distinguish LMS from other sarcoma types, except for poorly differentiated tumors, whereas UPS cannot be clearly classified based on its methylation profile [12].

Myostatin, a paracrine signaling molecule identified in 1997, belongs to the transforming growth factor-beta (TGF-β) superfamily. It is predominantly expressed and secreted by skeletal muscle, where it negatively regulates muscle growth through activin receptors [13]. Experimental overexpression of myostatin has been shown to cause skeletal muscle atrophy by downregulating muscle-specific gene expression [14]. In addition, myostatin promotes the degradation of muscle fibers, suppresses muscle development, induces atrophy, and decreases muscular strength [15,16,17]. In contrast, myostatin deficiency results in muscular hyperplasia or hypertrophy and promotes muscle growth [15,16,17].

In this study, we employed Cap Analysis of Gene Expression (CAGE) to explore the potential gene expression networks involved in the myogenic differentiation of PLMS relative to other high-grade sarcomas.

## 2. Results

### 2.1. CAGE Data Identified the MSTN Promoter as Being More Activated in High-Grade Sarcomas than in PLMS

**Figure 1:**
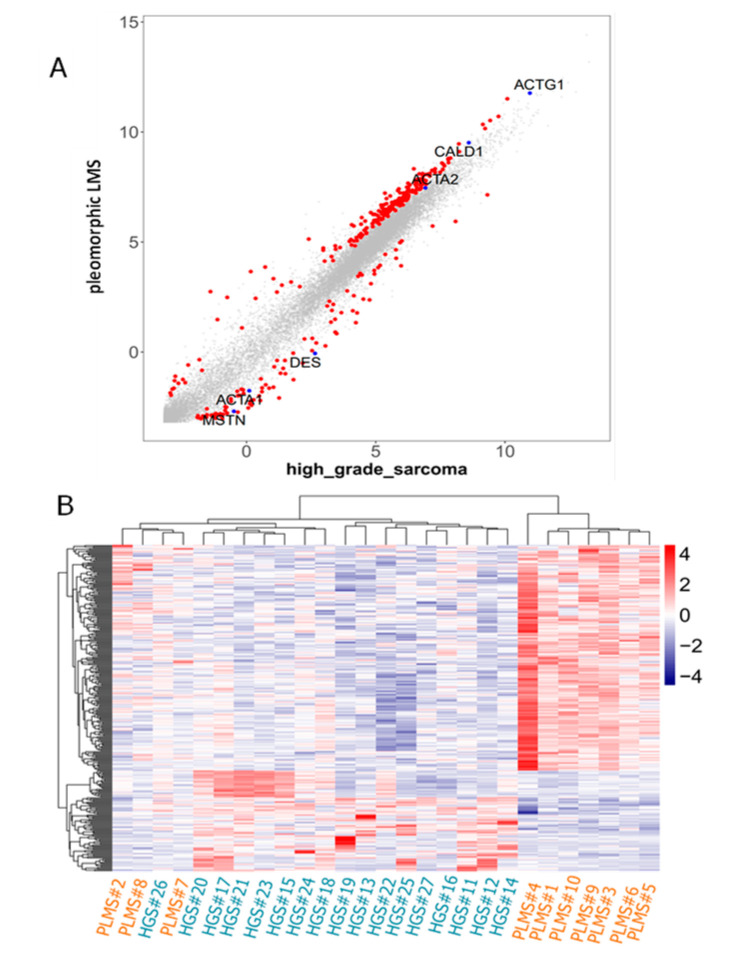
(**A**): Scatter plot of protein-coding genes (n = 15,402; gray dots) and differentially expressed genes (DEGs, n = 304; red dots). DEGs were identified via CAGE analysis. *MSTN* was significantly expressed in high-grade sarcomas compared with PLMS (FDR < 0.05). (**B**): Heatmap constructed using selected genes in all cases. A clustering analysis based on the 304 DEGs could separate 7 of the 10 PLMSs from other high-grade sarcomas, while the remaining three PLMSs formed a separate group with the single US case within high-grade sarcomas.

CAGE analysis revealed that, among 15,402 filtered genes, 304 differentially expressed genes (DEGs) were identified when comparing the PLMS and high-grade sarcoma groups. Analysis of the DEG promoter activity showed that the promoter of *MSTN*, which encodes myostatin, was more activated in the high-grade sarcoma group, although expression levels were low in both groups (Figure 1A). In addition, the promoter of *ACTA2*, which encodes alpha-smooth muscle actin, was more activated in the PLMS group. Furthermore, hierarchical clustering based on these DEGs separated 7 out of 10 PLMSs from other high-grade sarcomas, while the remaining three PLMSs clustered with the single undifferentiated sarcoma (US) case (Case 26) under the broader branch of high-grade sarcomas (Figure 1B).

Interestingly, this US case (Case 26) did not show myostatin expression in the IHC analysis and instead exhibited focal expression of myogenic markers. However, the typical fascicular arrangement of tumor cells—an important diagnostic criterion for LMS—was not observed (Table 1); therefore, the tumor was classified as a US. Myostatin has been shown to negatively regulate skeletal muscle growth via activin receptors. Based on this, we hypothesized that myostatin suppresses myogenic differentiation in high-grade sarcomas lacking smooth muscle differentiation and that reduced or absent myostatin expression may be associated with myogenic marker expression in PLMSs.

### 2.2. Myostatin and Myogenic Marker Expression in High-Grade Sarcomas and PLMSs

**Figure 2:**
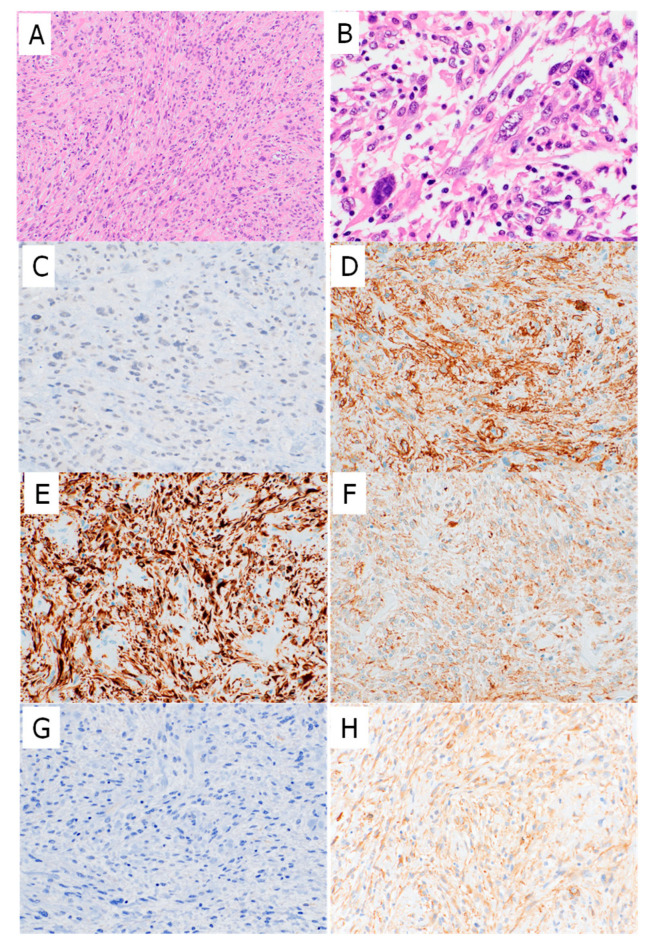
Microscopically, a case of pleomorphic leiomyosarcoma (PLMS) also contained a conventional LMS area, showing fascicular proliferation of spindle cells (**A**). along with a high-grade area composed of atypical spindle cells with enlarged hyperchromatic nuclei and abundant eosinophilic cytoplasm (**B**). Bizarre multinucleated tumor giant cells were also observed (**B**). Tumor cells were completely negative for myostatin (**C**). Tumor cells stained diffusely positive for SMA (**D**), desmin (**E**), M-actin (**F**), and l-caldesmon (**H**) but negative for h-caldesmon (**G**) (**A**, ×100; **B**, ×400; **C**–**H**, ×100).

**Figure 3:**
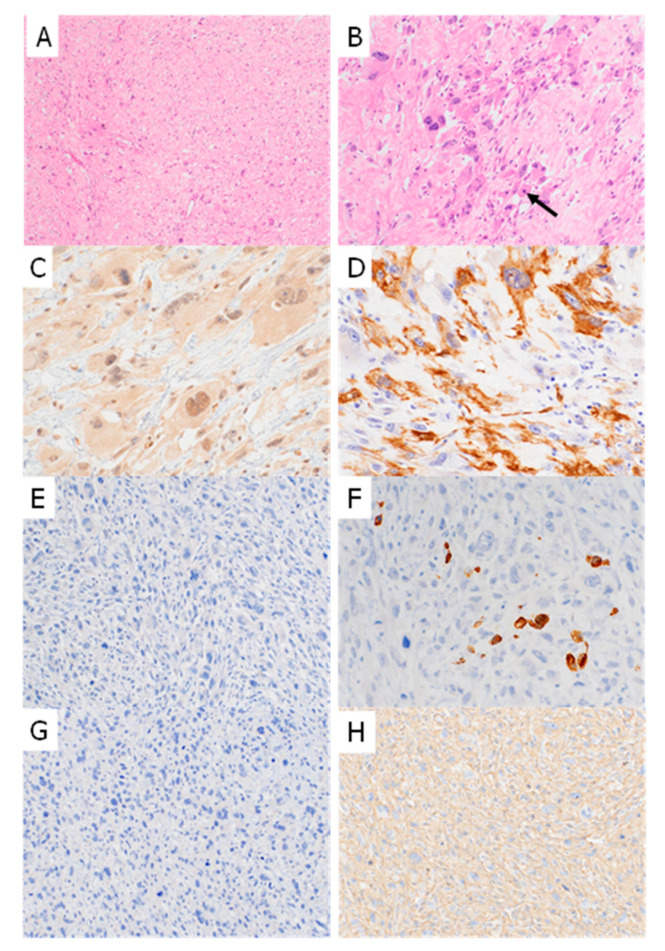
A case of undifferentiated pleomorphic sarcoma showing proliferation of spindle-shaped cells often having marked pleomorphism and eosinophilic cytoplasm. A fascicular proliferative pattern was not evident (**A**,**B**). A mitotic figure was observed (**B**: arrow). IHC shows diffuse myostatin positivity (**C**). Tumor cells were focally positive for SMA (**D**), negative for desmin (**E**), positive for M-actin (**F**), and negative for h-caldesmon (**G**). Tumor cells were also diffusely positive for l-caldesmon (**H**) (**A**, ×40; **B**,**E**–**H**, ×100; **C**,**D**, ×200).

Next, IHC was performed on the corresponding 27 formalin-fixed paraffin-embedded (FFPE) samples. The IHC findings are summarized in Table 1 and Table 2. Seven of the 10 PLMS cases (70%) were negative for myostatin expression (Figure 2 and Figure 3), while the remaining three cases showed either diffuse focal or focal myostatin expression (Table 1). Notably, two of these three myostatin-positive PLMS cases clustered together with high-grade sarcomas in the transcriptomic analysis (Figure 1B).

In contrast, 12 of the 17 high-grade sarcoma cases (71%) showed diffusely positive myostatin staining, and two (12%) showed focal staining. The overall myostatin positivity rate, therefore, differed between the PLMS and high-grade sarcoma groups. All PLMS cases were positive for smooth muscle actin (SMA); additionally, 7 (70%) and 6 (60%) of the 10 PLMS cases showed either diffuse or focal staining for M-actin and desmin, respectively. In contrast, 9 of the 10 PLMS cases (90%) were negative for h-caldesmon. An inverse correlation was observed between myostatin and SMA expression within the PLMS group (*p* = 0.02; Table 2). Among the high-grade sarcoma group, 4 of the 17 cases (23%) were diffusely positive for SMA, and 7 (41%) showed focal positivity. Eight cases were negative for desmin, and 11 were negative for M-actin. All 17 cases were negative for h-caldesmon. Significant inverse correlations were noted between myostatin expression and desmin (*p* = 0.032) and between myostatin and M-actin expression (*p* = 0.003; Table 2).

All but 1 of the 27 cases were positive for l-caldesmon, with the exception being a high-grade sarcoma case. Additionally, 6 of the 10 PLMS cases exhibited a myostatin-negative/desmin-positive (diffuse or focal) phenotype, while 7 of the 17 high-grade sarcomas displayed a myostatin-positive (diffuse)/desmin-negative (or very focally positive) phenotype.

Next, the inverse relationship between myostatin and myogenic markers was further validated in an independent cohort of 59 STSs (Table 3). Among the 16 sarcomas outside the LMS/PLMS group, 14 expressed myostatin, with diffuse expression observed in 11 cases. Of the five sarcomas that did not show diffuse myostatin expression, complete loss was seen in both pleomorphic rhabdomyosarcomas (PRMSs) (Figure S4), while the remaining three—each a high-grade myxofibrosarcoma (MFS)—showed focal or very focal expression (Figure S3). In contrast, loss of myostatin expression was observed in 17 of the 27 PLMSs (63%). Among the remaining cases, three showed very focal, three showed focal, and four showed diffuse myostatin expression. Furthermore, 10 of the 16 LMS cases (62.5%) lacked myostatin expression, while four showed very focal and two showed diffuse or weak expression. Diffuse myostatin expression was significantly more common in sarcomas outside the LMS/PLMS group than in the LMS/PLMS group (*p* < 0.001). The frequencies of myostatin expression were largely similar between PLMSs and LMSs. However, LMSs more frequently showed diffuse expression of SMA and M-actin than PLMSs (*p* = 0.0012 and 0.047, respectively). In PLMSs, myostatin and myogenic markers were histologically expressed in an almost mutually exclusive pattern (Figure S2 and Table 3). Additionally, myostatin expression was absent in all 10 angioleiomyomas examined (Figure S4).

The chemotherapeutic effects on the expression of myostatin and myogenic markers were assessable in three cases. In two PLMS cases (Cases 9 and 10), the expression status of these markers changed. Myostatin expression changed from focally positive to very focally positive in Case 9 and from negative to diffusely positive in Case 10, whereas the expression of myogenic markers was reinforced in both cases. Notably, one patient (Case 10) developed lung metastasis 7 months after surgery and died of the disease 17 months postoperatively, despite receiving neoadjuvant chemotherapy (Table 1). In another case (Case 27) of high-grade sarcoma, myostatin expression changed from very focally positive to diffusely positive, and focal expression of the myogenic marker M-actin emerged following chemotherapy.

*CALD1*, which encodes both h-caldesmon and l-caldesmon via alternative splicing, also emerged as a commonly activated gene in PLMSs and high-grade sarcomas. Therefore, IHC was performed to assess the expression of h-caldesmon—a smooth muscle marker—and l-caldesmon, which has been implicated in regulating proliferation and migration of vascular smooth muscle cells [18]. The results showed that h-caldesmon expression was focal to very focal in only 2 of the 10 PLMS cases and was entirely absent in the high-grade sarcoma cases. In contrast, l-caldesmon expression was observed in all but one of the high-grade sarcoma cases. Thus, activation of the *CALD1* promoter was found to reflect l-caldesmon expression and was not associated with myogenic differentiation. Regarding the other genes highlighted in Figure 1A, alpha-, beta-, and gamma-actins co-existed in most cell types as components of the cytoskeleton and mediators of internal cell motility, structure, and integrity. *ACTG1* encodes actin gamma 1, a cytoplasmic actin expressed in all cell types. Both *ACTA1* and *ACTG1* were highly expressed in the PLMS and high-grade sarcoma groups, with expression more pronounced in the latter. *ACTA2*, which encodes SMA, showed higher expression in the PLMS group. Desmin, encoded by *DES*, was expressed in both groups. IHC confirmed desmin expression in a substantial subset of PLMS and high-grade sarcoma samples.

### 2.3. Double Staining Showed Near-Inverse Expression Patterns of Myostatin and Myogenic Markers in High-Grade Sarcomas

**Figure 4:**
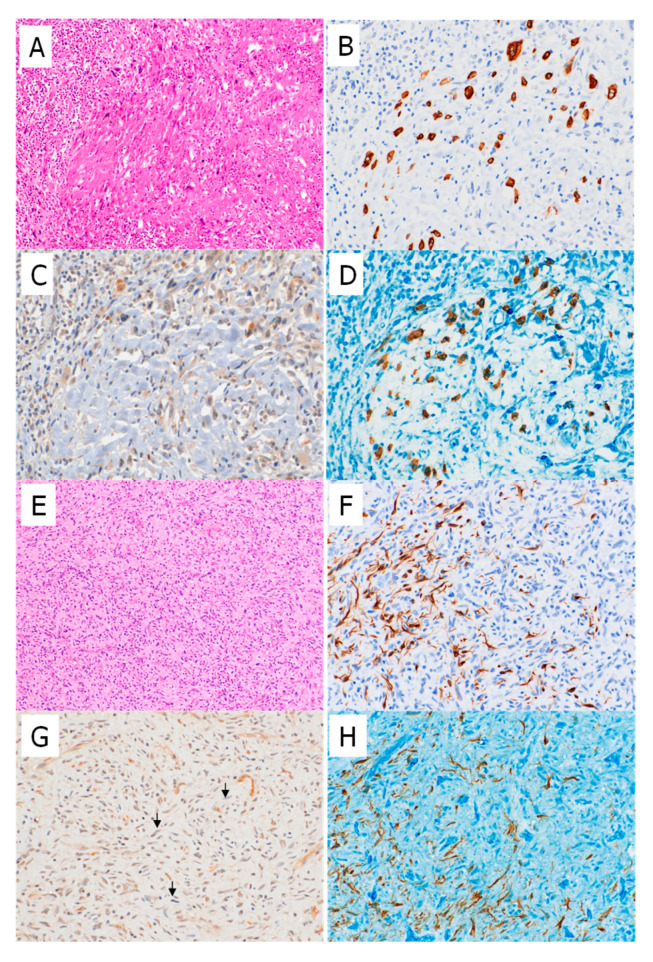
Double staining for myostatin and desmin in high-grade sarcomas without muscular differentiation. In a case of high-grade myxofibrosarcoma, an entrapped bundle of non-neoplastic skeletal muscles was present in the tumor center, along with massive inflammatory cell infiltration (**A**). Desmin staining was positive in non-neoplastic skeletal muscle fibers (**B**), while myostatin staining was negative in these fibers (**C**). Double staining for myostatin (blue) and desmin (brown) showed an almost mutually exclusive pattern of staining between neoplastic tumor cells and entrapped degenerative or atrophic skeletal muscle fibers (**D**). A case of dedifferentiated liposarcoma showing proliferation of spindle cells (**E**). Tumor cells with elongated cytoplasm were positive for desmin (**F**), while short spindle-shaped cells stained positively for myostatin, and cells with elongated cytoplasm were negative for myostatin (arrows) (**G**). Double staining for myostatin (blue) and desmin (brown) showed an almost mutually exclusive pattern (**H**) (**A**,**E**, ×100; **B**–**D**,**F**–**H**, ×200).

To examine whether myostatin and differentiation markers were mutually exclusive, double staining was performed in selected high-grade sarcoma samples that showed focal expression of both markers. Tumor cells lacking myostatin expression exhibited desmin or SMA expression, supporting the previously mentioned hypothesis (Figure 4).

## 3. Discussion

In STSs, specific areas within conventional tumors may occasionally exhibit overgrowth of dedifferentiated components. This phenomenon is also observed in well-differentiated liposarcomas (also termed atypical lipomatous tumors), resulting in dedifferentiated liposarcoma. PLMS is considered a variant of LMS. In immunohistochemical analyses, typical LMSs—characterized by muscle differentiation—commonly express myogenic markers. In contrast, these markers are often negative or decreased in the dedifferentiated regions of PLMSs. This pattern was confirmed in the present validation study, which showed that diffuse expression of SMA and M-actin was significantly more frequent in LMSs than in PLMS. Diffuse expression of desmin and h-caldesmon was also more frequently detected in LMSs, though the difference did not reach statistical significance.

Using unsupervised clustering in CAGE analysis revealed that PLMSs were largely distinct from other high-grade sarcomas without smooth muscle differentiation, indicating that PLMSs exhibit a unique gene expression profile. However, despite low overall expression in both groups, *MSTN*—a gene known to suppress muscle cell proliferation [19,20,21]—was more strongly activated in high-grade sarcomas than in PLMSs.

Myostatin, also known as growth/differentiation factor 8, is primarily expressed in skeletal muscle. It promotes muscle cell protein degradation and inhibits muscle cell growth, ultimately leading to muscle atrophy and weakness. Conversely, deletion of the myostatin gene induces myocyte hyperplasia and myofiber hypertrophy [15,16,17]. Myostatin inhibitors have therefore been explored as therapeutic agents for sarcopenia, a condition marked by reduced muscle mass, strength, and physical function. In general, IHC revealed that myostatin was frequently and diffusely expressed in high-grade sarcomas lacking myogenic differentiation, whereas its expression was less common in LMS and PLMSs. Conversely, myogenic markers were less frequently expressed in high-grade sarcomas without smooth muscle differentiation. Moreover, we confirmed via double staining that myostatin and desmin/SMA were expressed in the same tumor in an almost mutually exclusive pattern, even when myogenic markers were only focally expressed. Furthermore, the validation study showed no difference in myostatin expression rate between LMSs and PLMSs. These findings suggest that reduced or absent myostatin expression may be associated with myogenic differentiation but not with plemorphism among high-grade sarcomas.

Previous studies have shown that, morphologically and immunohistochemically, poorly differentiated (pleomorphic) LMSs may mimic undifferentiated sarcomas due to a progressive loss of muscle markers [22]. Consistent with this, we also observed higher frequencies of myogenic marker expression—such as SMA and M-actin—in LMSs than in PLMSs. In addition, even when myogenic markers were heterogeneously expressed, their presence was almost always associated with an absence of myostatin expression. This finding was also validated in the current study cohort. Furthermore, gene expression profiling stratified LMSs into three molecular subtypes [23]. Among these, LMS Group 1 (primarily comprising conventional LMSs) demonstrated significantly enriched expression of genes involved in muscle contraction and actin cytoskeleton organization, such as *CALD1* and *ACTG2.* This group also showed a significantly better prognosis than the other two groups, which predominantly comprised PLMS exhibiting less myogenic differentiation than conventional LMSs [24]. Considering that the progressive loss of myogenic characteristics in LMS is associated with adverse outcomes, it is hypothesized that myostatin expression may similarly affect the prognosis of patients with PLMS. This hypothesis raises several important questions: What is the prognosis of PLMS based on myostatin expression? Is myostatin expression associated with chemoresistance? Can tumor cells acquire myostatin expression during chemotherapy? Regarding the first question, two of the three PLMS cases with focal myostatin expression experienced recurrence, as did five of the seven PLMS cases lacking myostatin expression (Table 1). Therefore, no association between myostatin expression and prognosis could be established, although the sample size in this study was limited.

Interestingly, myostatin has also been shown to regulate epithelial–mesenchymal transition genes and enhance the invasiveness of human trophoblast cells [25]. Thus, further studies involving larger samples are needed. No definitive conclusions could be drawn regarding the second question, as four of the five patients with PLMS who received chemotherapy developed distant metastases in this series. Post-chemotherapy histological evaluation was available for three cases; however, no definitive conclusions could be made regarding the relationship between myostatin expression and chemotherapeutic response (Table 1). Notably, myostatin expression was enhanced in two of the three cases after chemotherapy, while the expression of myogenic markers was upregulated in all three. These findings suggest that chemotherapy may influence IHC protein expression profiles and even the diagnosis of STSs. Nevertheless, further studies are required to answer these questions.

CAGE analysis also demonstrated that the high-grade sarcomas examined were enriched for activated promoters of genes involved in immune system pathways (Figure S5). In this cohort, 11 of the 17 high-grade sarcomas were high-grade MFSs—a subtype characterized, in part, by lymphoplasmacytic infiltration. Therefore, the CAGE results may have been largely influenced by this histologic subtype.

Yamashita et al. reported a high number of tumor-infiltrating lymphocytes in high-grade MFS and proposed immune checkpoint inhibitors as potential therapeutic targets in a subset of these tumors [26], aligning with our findings. In contrast, the lack of clinical benefit of PD-1 inhibitors in patients with LMS is well-documented [27,28], and a recent study confirmed that combination therapy with lenvatinib and pembrolizumab did not overcome this primary resistance [29]. These studies also support our observation that genes involved in immune system pathways are not activated in PLMS compared to other high-grade sarcomas. From the perspective of identifying new and effective therapies for LMS, our recent studies suggest that many potential therapeutic targets may remain undiscovered. Notably, among various high-grade sarcoma subtypes, tyrosine kinase fusions—specifically *ROS1* and *NTRK3*—have been detected only in LMS cases [30,31]. Beyond the role of myogenic differentiation, loss of myostatin expression may serve as a marker for sarcomas harboring potential therapeutic targets.

This study has a few limitations. First, the CAGE samples were obtained at the time of biopsy; therefore, the proportions of conventional and pleomorphic components in the PLMS cases were unknown, potentially affecting CAGE results. Second, the sample size was limited, precluding definitive conclusions and highlighting the need for larger-scale studies.

Finally, we found that the *MSTN* promoter was activated in high-grade sarcomas lacking both morphological and immunohistochemical evidence of smooth muscle differentiation. Although myostatin staining was not strong, this finding reflected the relatively low expression level of *MSTN* in sarcoma samples observed via CAGE, despite its comparatively higher expression in high-grade sarcomas than in PLMS. Myostatin expression, encoded by *MSTN,* was almost inversely correlated with the expression of myogenic markers, suggesting that it may play a role in smooth muscle differentiation in high-grade sarcomas. Even at low expression levels, myostatin may be associated with the loss of smooth muscle differentiation in these tumors. Previous reports have noted that the expression levels of myogenic markers tend to be lost in PLMS, especially in high-grade areas [23], complicating the diagnosis. Although this study did not aim to identify specific diagnostic markers for PLMS, the absence of myostatin expression on IHC may help differentiate PLMS from other high-grade sarcomas and support its diagnosis.

## 4. Materials and Methods

### 4.1. Samples

We selected 27 high-grade sarcomas from the pathology archives of the Pathology Department of Juntendo University Hospital, Tokyo, Japan, for which fresh frozen material was available for CAGE. The samples included 10 PLMSs, 11 high-grade MFSs, 3 dedifferentiated liposarcomas, 2 USs, and 1 high-grade not otherwise specified (NOS) sarcoma. Clinicopathologic information, including survival data, was obtained for all patients. Samples were obtained either at biopsy or surgical resection from treatment-naïve tumors, except for one case (Case 27), in which the sample was collected after preoperative chemotherapy. Postoperative chemotherapy was administered in nine cases, and preoperative neoadjuvant chemotherapy was administered in three cases (Cases 9, 10, and 27). The diagnosis of PLMS was based on the WHO classification of tumors [6]. All PLMS samples contained conventional leiomyosarcomatous fascicular areas in addition to pleomorphic regions. Immunohistochemically, all 10 PLMS cases in this study showed positive reactivity for at least two smooth muscle markers: desmin, muscle-specific actin (M-actin), alpha-smooth muscle actin (α-SMA), and h-caldesmon. Furthermore, to validate findings from CAGE and IHC on FFPE tissue, an additional cohort of 59 STSs and 10 angioleiomyomas was analyzed. The sarcoma cases included 27 PLMS, 16 LMS, 6 high-grade MFS, 4 USs, 2 PRMS, 1 intermediate-grade MFS, 1 low-grade MFS, 1 low-grade fibromyxoid sarcoma, and 1 low-grade NOS sarcoma. The study was reviewed and approved by the Institutional Review Board of the Juntendo University School of Medicine (approval number: #2021-079).

### 4.2. CAGE

We analyzed promoter activity profiles in the 27 high-grade sarcoma samples. All samples submitted for CAGE analysis were derived from tumors with no prior history of chemoradiation, except for Case 27, which had received preoperative chemotherapy. RNA was extracted from the fresh frozen samples using the RNeasy Plus Mini Kit (QIAGEN, Hilden, Germany). CAGE library preparation and sequencing were performed at K.K.DNAFORM. Adapter sequences were trimmed using Trimmomatic v0.39 (Trimmomatic: https://pubmed.ncbi.nlm.nih.gov/24695404/ (accessed on 1 March 2020)). The resulting reads were aligned to the human rDNA genome (accessions U13369.1 and V00589.1) using STAR v2.7.10a, with the parameters outFilterMismatchNmax set to 10 and outFilterMultimapNmax set to 500. Reads not mapped to the rDNA genome were subsequently aligned to the GRCh38 reference genome (primary assembly) using GENCODE v41 annotations (primary assembly) with STAR v2.7.10a. Uniquely mapped reads were extracted using SAMtools v1.15.1 (SAMtools: https://pubmed.ncbi.nlm.nih.gov/19505943/ (accessed on 1 March 2020)). The 5′ ends of the uniquely mapped reads were counted using bedGraphtobigWig for promoter regions, defined as transcription start sites ± 300 base pairs of protein-coding genes in GENCODE v41 [32,33]. Promoter count data were collapsed to gene-level counts, normalized as counts per million (CPM), and analyzed for differential expression using edgeR v3.34.0 (R v4.1.0). Lowly expressed promoters were filtered out using filterByExpr (min.count = 1). Genes with a FDR < 0.05 were defined as DEGs. Gene Ontology (GO) enrichment analysis of the DEGs was performed using the Metascape (http://metascape.org (accessed on 1 March 2020)). Relevant preprocessing scripts for CAGE data are available at https://github.com/MurakawaLab/CAGE_pipeline (accessed on 1 March 2020).

### 4.3. Immunohistochemical Staining

We used FFPE tissue blocks of surgically resected sarcomas corresponding to the frozen samples used for CAGE analysis. These were fixed in 10% buffered formalin after routine processing. For the three cases that received neoadjuvant chemotherapy, therapy-naïve biopsy samples were also examined. Four-micrometer-thick sections were stained using the six antibodies listed in Supplementary Table S1. Two uterine adenomyosis samples from Juntendo University Hospital were used as positive controls for myostatin IHC (Figure S1). In addition, two samples obtained from cases that had received chemotherapy (Cases 10 and 26) were analyzed. Immunohistochemical staining was evaluated under a microscope and classified as follows: diffusely positive (staining in more than half of the sample area), focally positive (staining in one-third to one-half of the sample area), and very focally positive (staining in less than one-third of the sample area). For SMA, M-actin, desmin, and h-caldesmon, either cytoplasmic or membranous staining was interpreted as positive. For myostatin, both cytoplasmic and nuclear staining were considered indicative of positivity.

### 4.4. Double Staining

We performed immunohistochemical double staining to confirm the distribution and co-localization patterns of myostatin and myogenic markers. A primary antibody cocktail containing antibodies against myostatin and either desmin or SMA was used. The secondary antibodies were alkaline phosphatase-conjugated goat anti-rabbit and horseradish peroxidase-conjugated goat anti-mouse antibodies (MACH2 Double Stain 2 Mouse-HRP + Rabbit-AP Polymer Detection Kit, BioCare Medical, CA, USA).

## 5. Conclusions

The expression of myostatin, encoded by *MSTN*, may play a role in the regulation of smooth muscle differentiation in PLMS.

## Figures and Tables

**Table 1:** Clinicopathological and immunohistochemical findings in CAGE cases.

Case No.	Histological Type	Material	Myostatin	SMA	Desmin	M−Actin	h−Caldesmon	l−Caldesmon	Chemotherapy	Recurrence/Metastasis	Recurrence/Metastatic Site	Prognosis	Overall Survival (Months)	Chemotherapeutic Effect (%)
1	Pleomorphic leiomyosarcoma	resection	(−)	(focally +)	(focally +)	(diffuse +)	(−)	(diffuse +)	(+)	(+)	Lung (46 months)	DOD	DOD 58	
2	Pleomorphic leiomyosarcoma	resection	(−)	(diffuse +)	(diffuse +)	(diffuse +)	(−)	(diffuse +)	(−)	(−)		Alive	NED 68	
3	Pleomorphic leiomyosarcoma	resection	(−)	(focally +)	(focally +)	(−)	(−)	(diffuse +)	(+)	(+)	Lung (7 months)	Alive	AWD 60	
4	Pleomorphic leiomyosarcoma	resection	(−)	(diffuse +)	(focally +)	(focally +)	(focally +)	(diffuse +)	(−)	(−)		Alive	NED 53	
5	Pleomorphic leiomyosarcoma	resection	(−)	(focally +)	(focally +)	(−)	(−)	(focally +)	(−)	(+)	Lung (3 months)	DOD	DOD 5	
6	Pleomorphic leiomyosarcoma	resection	(−)	(diffuse +)	(−)	(diffuse +)	(−)	(diffuse +)	(−)	(+)	Lung/bone (41 months)	Alive	AWD 47	
7	Pleomorphic leiomyosarcoma	resection	(focally +)	(focally +)	(−)	(focally +)	(−)	(diffuse +)	(−)	(+)	Lung/chest wall/lymph node (19 months)	Alive	AWD 28	
8	Pleomorphic leiomyosarcoma	resection	(focally +)	(focally +)	(−)	(focally +)	(−)	(diffuse +)	(+)	(−)		Alive	NED 31	
9	Pleomorphic leiomyosarcoma	biopsy	(focally+)	(diffuse +)	(−)	(diffuse +)	(−)	(diffuse +)	Neoadjuvant (+)	(+)	Lung (8 months)	Alive	AWD 81	20
9-#2 *	Pleomorphic leiomyosarcoma	resection	(very focally+)	(diffuse +)	(focally +)	(diffuse +)	(very focally+)	(diffuse +)						
10	Pleomorphic leiomyosarcoma	biopsy	(−)	(very focally +)	(focally +)	(−)	(−)	(focally +)	Neoadjuvant (+)	(+)	Lung (9 months)	DOD	DOD 17	0
10-#2 *	Pleomorphic leiomyosarcoma	resection	(diffuse +)	(diffuse +)	(focally +)	(diffuse +)	(focally +)	(diffuse +)						
11	High−grade myxofibrosarcoma	resection	(focally +)	(diffuse +)	(diffuse +)	(diffuse +)	(−)	(diffuse +)						
12	Dedifferentiated Liposarcoma	resection	(diffuse +)	(focally +)	(focally +)	(−)	(−)	(diffuse +)						
13	Dedifferentiated Liposarcoma	resection	(diffuse +)	(−)	(focally +)	(−)	(−)	(focally +)						
14	Dedifferentiated Liposarcoma	resection	(diffuse +)	(diffuse +)	(diffuse +)	(−)	(−)	(focally +)						
15	High−grade myxofibrosarcoma	resection	(diffuse +)	(−)	(−)	(−)	(−)	(focally +)						
16	High-grade myxofibrosarcoma	resection	(diffuse +)	(diffuse +)	(−)	(diffuse +)	(−)	(diffuse +)						
17	High-grade myxofibrosarcoma	resection	(diffuse +)	(diffuse +)	(−)	(−)	(−)	(diffuse +)						
18	High-grade myxofibrosarcoma	resection	(−)	(−)	(−)	(−)	(−)	(−)						
19	High-grade myxofibrosarcoma	resection	(diffuse +)	(−)	(−)	(−)	(−)	(diffuse +)						
20	High-grade myxofibrosarcoma	resection	(diffuse +)	(focally +)	(diffuse +)	(−)	(−)	(diffuse +)						
21	High-grade myxofibrosarcoma	resection	(focally +)	(−)	(diffuse +)	(focally +)	(−)	(diffuse +)						
22	High-grade myxofibrosarcoma	resection	(diffuse +)	(−)	(−)	(−)	(−)	(diffuse +)						
23	High-grade myxofibrosarcoma	resection	(diffuse +)	(focally +)	(very focally +)	(diffuse +)	(−)	(diffuse +)						
24	High-grade myxofibrosarcoma	resection	(diffuse +)	(focally +)	(focally +)	(−)	(−)	(diffuse +)						
25	High-grade sarcoma, NOS	resection	(diffuse +)	(focally +)	(very focally +)	(very focally +)	(−)	(diffuse +)						
26	Undifferentiated sarcoma	resection	(−)	(focally +)	(−)	(focally +)	(−)	(diffuse +)						
27	Undifferentiated sarcoma	biopsy	(very focally +)	(focally +)	(−)	(−)	(−)	(diffuse +)						
27-#2 *	Undifferentiated sarcoma	resection	(diffuse +)	(diffuse +)	(−)	(focally +)	(−)	(diffuse +)						

Abbreviations: SMA, smooth muscle actin; M-actin, muscle actin; DOD, died of disease; AWD, alive with disease; NED, no evidence of disease; OS, overall survival; NOS, not otherwise specified; (+), positive; (−), negative; * #2 sample: Corresponding surgically resected sample after chemotherapy.

**Table 2:** Positive rate for each antibody in pleomorphic leiomyosarcoma and high-grade sarcoma (FFPE cases).

Histological Type	IHC Expression	Myostatin	SMA	Desmin	M-Actin	h-Caldesmon	l-Caldesmon
Pleomorphic leiomyosarcoma	(−)	7 (70%)	0 (0%)	4 (40%)	3 (30%)	9 (90%)	0 (0%)
N = 10	(very focally +)	0 (0%)	1 (10%)	0 (0%)	0 (0%)	0 (0%)	0 (0%)
	(focally +)	3 (30%)	5 (50%)	5 (50%)	3 (30%)	1 (10%)	2 (20%)
	(diffuse +)	0 (0%)	4 (40%)	1 (10%)	4 (40%)	0 (0%)	8 (80%)
		*p* value	0.02	0.37	0.18	0.58	0.003
High-grade sarcoma	(−)	2 (11%)	6 (35%)	8 (47%)	11 (64%)	17 (100%)	1 (5%)
N = 17	(very focally +)	1 (5%)	0 (0%)	2 (11%)	1 (5%)	0 (0%)	0 (0%)
	(focally +)	2 (11%)	7 (41%)	3 (17%)	2 (11%)	0 (0%)	3 (17%)
	(diffuse +)	12 (70%)	4 (23%)	4 (23%)	3 (17%)	0 (0%)	13 (76%)
		*p* value	0.28	0.032	0.003	0	0.6

*p* value: Correlation between myostatin expression and myogenic markers. Statistical analysis was performed comparing the following subgroups: (−) and (very focally +) vs. (focally +) and (diffuse +). Abbreviations: IHC, immunohistochemistry; SMA, smooth muscle actin; M-actin, muscle actin; FFPE, formalin-fixed paraffin-embedded.

**Table 3:** Validation of immunohistochemistry results in sarcoma cases.

	Diagnosis	Myostatin	SMA	Desmin	M-Actin	h-Caldesmon
V#1	US	diffuse +	focal +	(−)	very focal +	(−)
V#2	US	diffuse +, weak	−	−	−	−
V#3	US	diffuse +	very focal +	(−)	(−)	(−)
V#4	US	diffuse +	very focal +	(−)	(−)	(−)
V#5	Low-grade FMS	diffuse +	−	−	−	−
V#6	Low-grade sarcoma, NOS	diffuse +	very focal +	(−)	(−)	(−)
V#7	Low-grade MFS	diffuse +	−	−	−	−
V#8	Intermediate-grade MFS	diffuse +	−	−	−	−
V#9	High-grade MFS	diffuse +	−	−	−	−
V#10	High-grade MFS	diffuse +	−	−	−	−
V#11	High-grade MFS	focal +	focal +	−	−	−
V#12	High-grade MFS	very focal +, weak	focal +	−	−	−
V#13	High-grade MFS	focal +, weak	focal +	−	−	−
V#14	High-grade MFS	diffuse +	−	−	−	−
V#15	PRMS	(−)	very focal +	diffuse +	diffuse +	−
V#16	PRMS	(−)	very focal +	diffuse +	−	−
V#17	PLMS	focal +, strong	diffuse +	diffuse +	diffuse +	diffuse +
V#18	PLMS	very focal +	diffuse +	focal +	diffuse +	diffuse +
V#19	PLMS	(−)	very focal +	−	−	−
V#20	PLMS	(−)	focal +	−	very focal +	−
V#21	PLMS	diffuse +, weak	diffuse +	−	focal+	very focal +
V#22	PLMS	diffuse +, weak	very focal +	diffuse +	very focal +	−
V#23	PLMS	very focal +, weak	−	−	−	−
V#24	PLMS	(−)	very focal +	very focal +	−	−
V#25	PLMS	(−)	diffuse +	very focal +	focal +	−
V#26	PLMS	(−)	focal +	−	−	−
V#27	PLMS	(−)	focal +	focal +	very focal +	−
V#28	PLMS	(−)	diffuse +	diffuse +	diffuse +	diffuse +
V#29	PLMS	(−)	very focal +	−	−	−
V#30	PLMS	(−)	focal +	very focal +	−	−
V#31	PLMS	focal +, weak	very focal +	−	−	−
V#32	PLMS	(−)	focal +	−	−	−
V#33	PLMS	very focal +, weak	diffuse +	−	focal +	−
V#34	PLMS	focal +, weak	diffuse +	diffuse +	diffuse +	diffuse +
V#35	PLMS	(−)	very focal +	−	−	very focal +
V#36	PLMS	(−)	diffuse +	focal +	very focal +	very focal +
V#37	PLMS	(−)	very focal +	−	−	−
V#38	PLMS	(−)	focal +	−	focal +	−
V#39	PLMS	(−)	focal +	−	focal +	−
V#40	PLMS	(−)	very focal +	focal +	−	−
V#41	PLMS	(−)	−	focal +	−	−
V#42	PLMS	diffuse +, weak	diffuse +	diffuse +	diffuse +	diffuse +
V#43	PLMS	diffuse +, weak	very focal +	very focal +	very focal +	−
V#44	LMS	diffuse +, weak	diffuse +	diffuse +	diffuse +	diffuse +
V#45	LMS	(−)	diffuse +	diffuse +	diffuse +	diffuse +
V#46	LMS	(−)	diffuse +	−	−	−
V#47	LMS	(−)	diffuse +	diffuse +	diffuse +	diffuse +
V#48	LMS of bone	(−)	focal +	−	−	−
V#49	LMS	very focal +	diffuse +	very focal +	diffuse +	diffuse +
V#50	LMS	(−)	diffuse +	focal +	very focal+	−
V#51	LMS	(−)	focal +	diffuse +	diffuse +	diffuse +
V#52	LMS	(−)	diffuse +	focal +	diffuse +	diffuse +
V#53	LMS	(−)	diffuse +	diffuse +	diffuse +	diffuse +
V#54	LMS	diffuse +, weak	diffuse +	−	focal+	−
V#55	LMS	very focal +, weak	diffuse +	−	diffuse +	−
V#56	LMS	very focal +, weak	diffuse +	−	focal +	−
V#57	LMS	(−)	diffuse +	very focal +	focal +	−
V#58	LMS	(−)	diffuse +	−	focal +	−
V#59	LMS	very focal +, weak	diffuse +	very focal +	focal +	−

US, undifferentiated sarcoma; FMS, fibromyxoid sarcoma; MFS, myxofibrosarcoma; PRMS, pleomorphic rhabdomyosarcoma; PLMS, pleomorphic leiomyosarcoma; LMS, leiomyosarcoma; SMA, smooth muscle actin; M-actin, muscle actin.

## Data Availability

The data supporting the findings of this study are not publicly available because they contain information that could compromise the privacy of the research participants. However, the data is available from the corresponding author (T.S.) upon reasonable request.

## Supplementary Materials

The following supporting information can be downloaded at: https://www.mdpi.com/article/10.3390/ijms26167676/s1.
