# Supplementary material for: Potential Involvement of Myostatin in Smooth Muscle Differentiation in Pleomorphic Leiomyosarcoma
Source: Int J Mol Sci. 2025 Aug 8;26(16):7676. doi: 10.3390/ijms26167676 (PMC12386979; doi:10.3390/ijms26167676)

Supplementary Figure S1

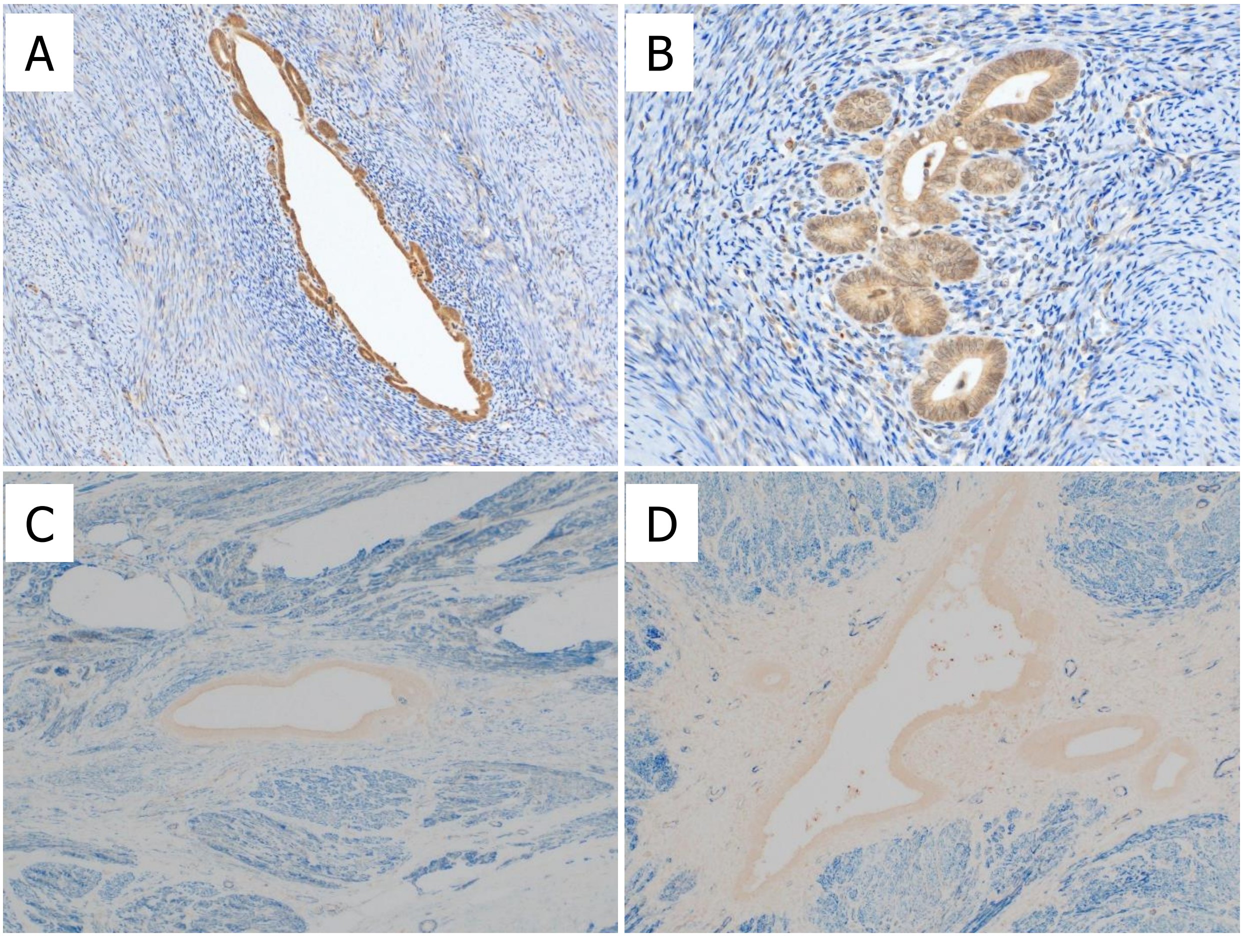

Supplementary Figure S2

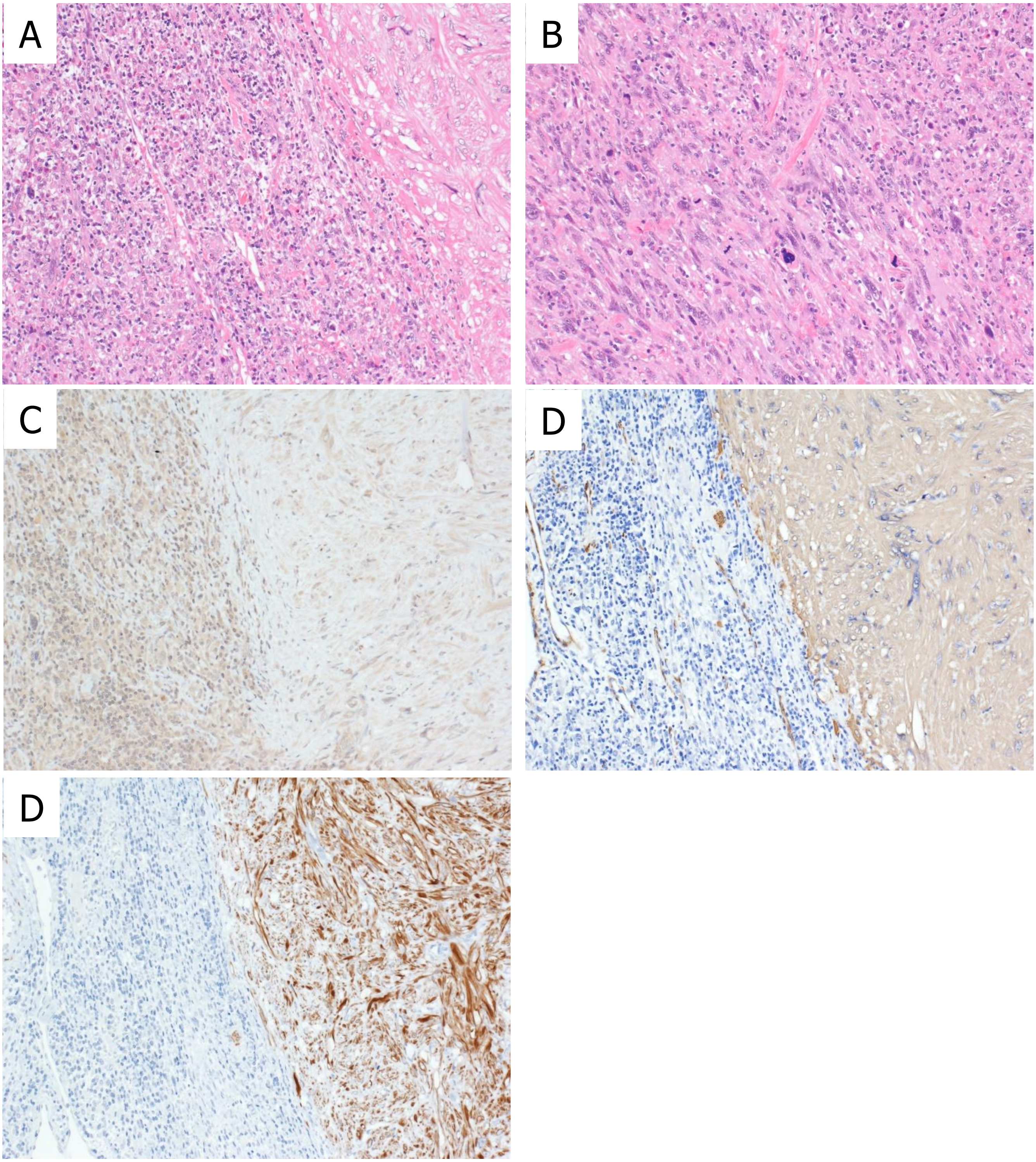

Supplementary Figure S3

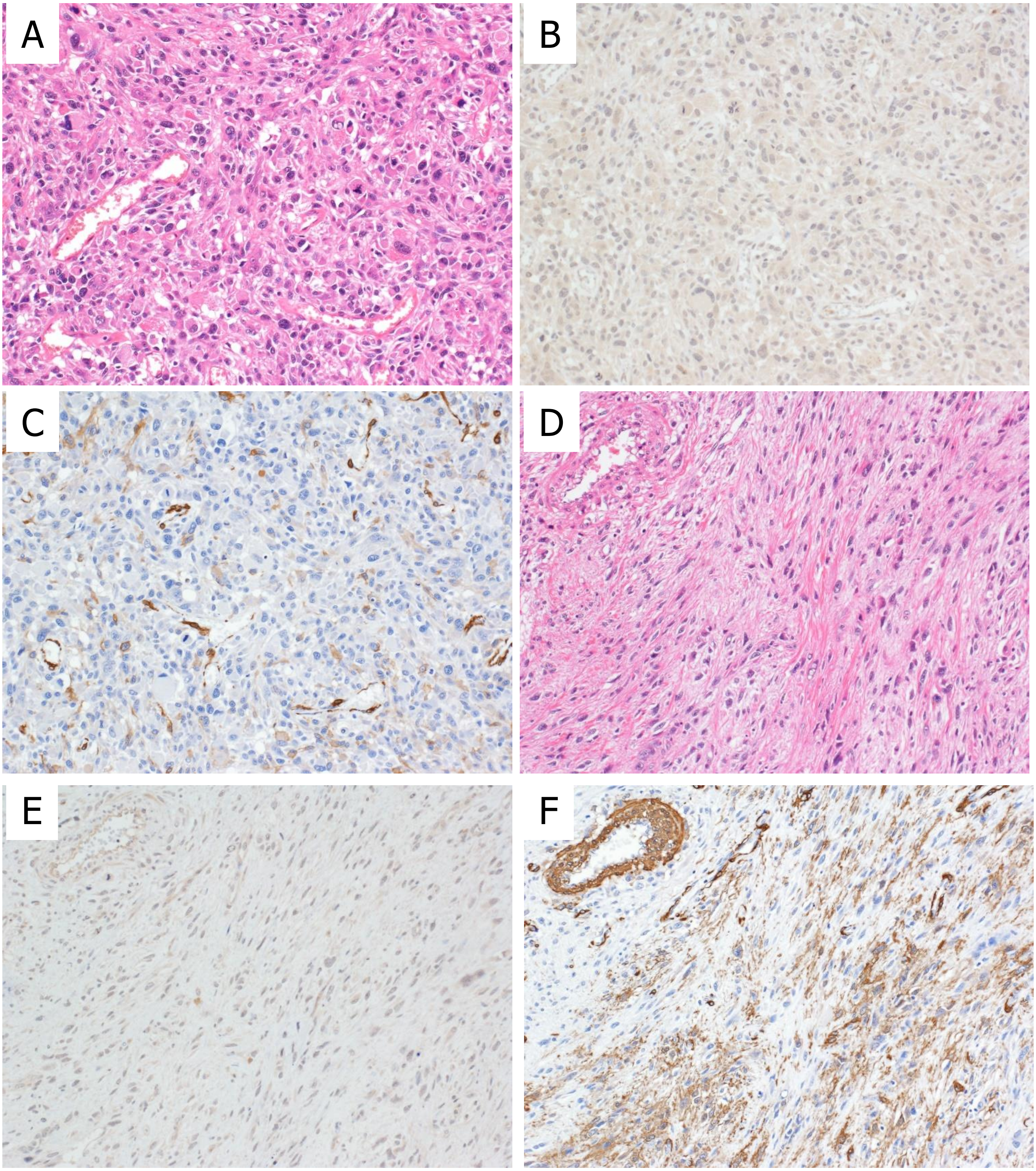

Supplementary Figure S4

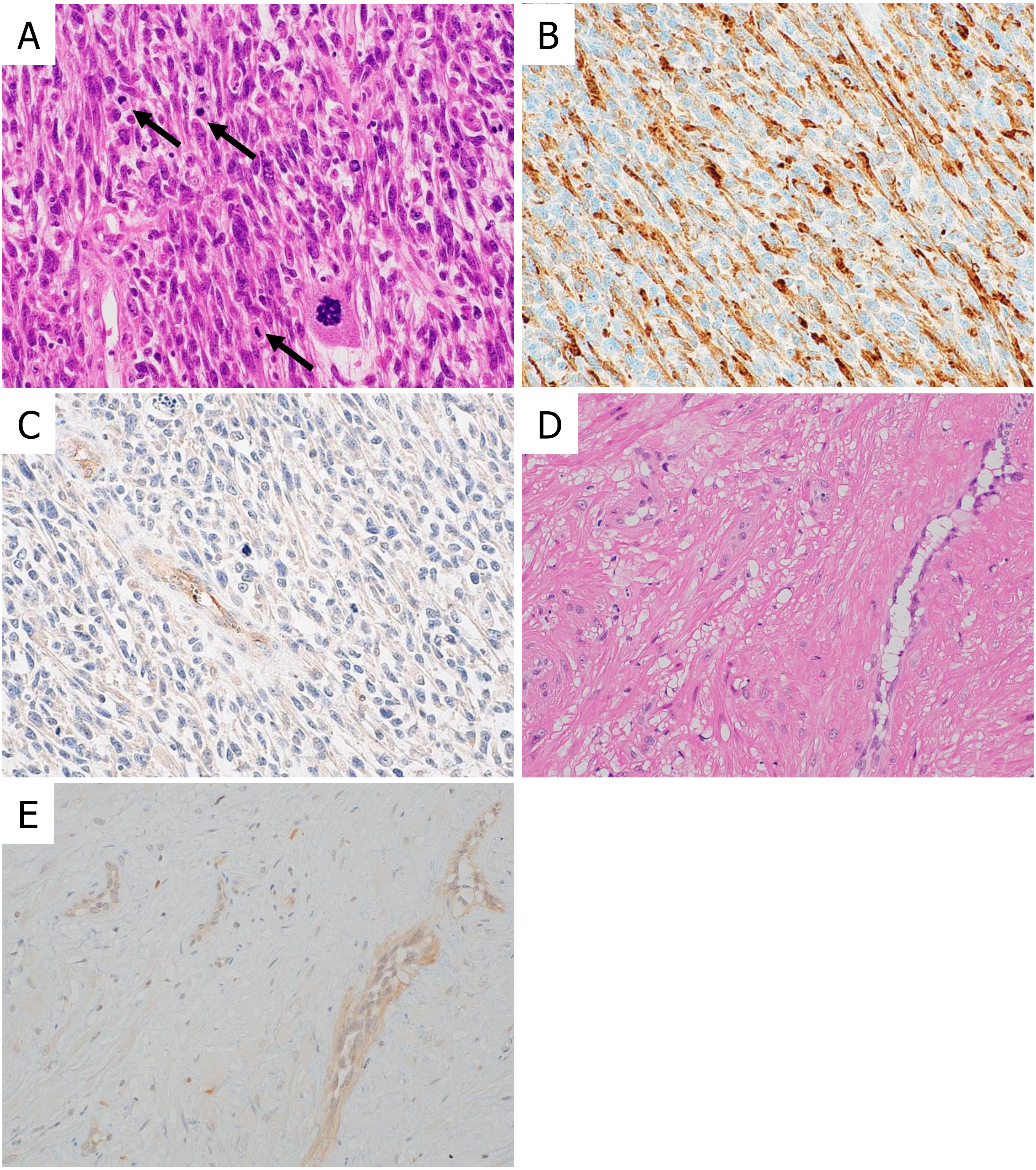

Supplementary Figure S5

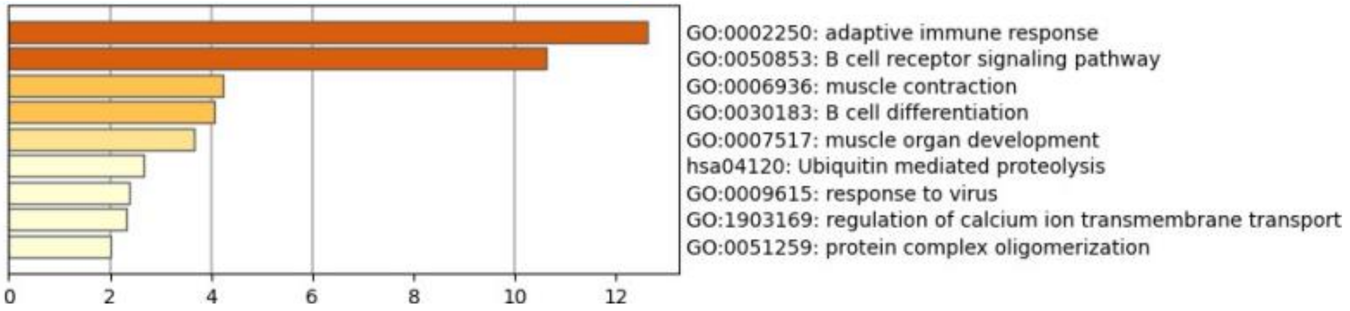

Supplement: Supplementary file 1 [file ijms-26-07676-s001.zip › ijms-3431260-supplementary.pdf]
