# Supplementary material for: Potential Involvement of Myostatin in Smooth Muscle Differentiation in Pleomorphic Leiomyosarcoma
Source: Int J Mol Sci. 2025 Aug 8;26(16):7676. doi: 10.3390/ijms26167676 (PMC12386979; doi:10.3390/ijms26167676)
Supplement: Supplementary file 1 [file ijms-26-07676-s001.zip › Supplementary Figure legends 08072025.pdf]

Figure S1: Two uterine adenomyosis samples were used as positive controls for myostatin IHC. Myostatin expression (brown) was observed in the glandular cells but not in the muscular layer (A, B). Double immunostaining for myostatin (brown) and SMA (blue) showed that glandular cells in the adenomyosis sample were positive for myostatin but negative for SMA, whereas smooth muscle cells were positive for SMA but negative for myostatin (C, D). Panels A and C are from one case, and panels B and D are from another (A, B x100; C, D x200).

Figure S2: Myostatin, SMA, and desmin expression in a case of PLMS (V#17 in Table 3). A: Borderline between conventional LMS area (right) and dedifferentiated high-grade area (left). B: High-power view of the high-grade area with frequent mitotic figures. C: Myostatin expression was observed in the high-grade area (left) but not in the conventional area (right). SMA (D) and desmin (E) expression was observed in the conventional area (right) but not in the high-grade area (left).

Figure S3: Myostatin and SMA expression in a high-grade MFS case (V#12in Table 3). A high-grade area (A) showed weak myostatin expression (B) but no SMA expression (C) in tumor cells. An intermediate-grade area (D) showed myostatin expression in the vascular endothelium but not in tumor cells (E), whereas SMA expression was observed in tumor cells (F).

Figure S4: Myostatin expression in benign and malignant tumors with myogenic differentiation.

(A) Case of pleomorphic rhabdomyosarcoma containing tumor giant cells admixed with rhabdomyoblasts; mitotic figures are frequently seen (arrows). (B) Tumor cells were desmin-positive and (C) myostatin-negative, although vascular endothelium was positive for myostatin. (D, E) In angioleiomyoma, short spindle cells were myostatin-negative, while

vascular endothelial cells were myostatin-positive (A–E: x200).

Figure S5: Gene Ontology (GO) enrichment analysis revealed that among DEGs, genes related to immune system pathways were highly expressed in high-grade sarcomas.
